# Paraphrenia – current psychopathological and diagnoses landmarks

**DOI:** 10.1192/j.eurpsy.2024.974

**Published:** 2024-08-27

**Authors:** E. Lukacs, A. Nirestean, L. M. Grebenisan, A. Sima Comaniciu, E. G. Strete

**Affiliations:** Psychiatry, George Emil Palade University of Medicine, Pharmacy, Science, and Technology of Targu Mures, Targu Mures, Romania

## Abstract

**Introduction:**

Paraphrenia, classically known as a chronic delusional-hallucinatory psychosis, currently has an uncertain nosological status, not being included in DSM-5 either. It can be integrated into the group of schizophrenic and delusional psychoses, but with obvious distinctive attributes. Currently, in the context of the increase in the incidence of childhood autism, the psychopathological pictures from the spectrum of psychoses in adulthood are also diversifying. Paraphrenic clinical pictures retain their specificity regarding the subject’s functioning in life roles and the absence of cognitive impairment despite the absurdity of delusional ideas while maintaining a good insertion in reality.

**Objectives:**

We refer to patients who can be classically classified in the diagnosis of paraphrenia, with the aim of bringing back into question the validity and authenticity of this nosological entity.

**Methods:**

The case descriptions aim to highlight the common clinical-evolutionary attributes and the distinctive ones between paraphrenia and other schizophrenic and delusional psychoses, emphasizing the differentiations corresponding to the involvement of personality and the ability to function in life roles.

**Results:**

It is confirmed that in the case of subjects who can be classified as paraphrenic, fundamental personality structures are preserved, a good adaptation in roles with insignificant cognitive deterioration phenomena, a well-preserved insight but with a high potential of unpredictability so characteristic of the world of psychoses.

**Conclusions:**

We believe that paraphrenia remains a psychopathological and clinical entity within which, although opposites coexist, the reporting and adaptation to objective reality is preserved - thanks to “double accounting”. From this perspective, paraphrenia confirms its distinct nosological status.

**Disclosure of Interest:**

None Declared

